# Supplementary material for: Correlation between Lindane Use and the Incidence of Thyroid Cancer in the United States: An Ecological Study
Source: Int J Environ Res Public Health. 2022 Oct 13;19(20):13158. doi: 10.3390/ijerph192013158 (PMC9602460; doi:10.3390/ijerph192013158)
Supplement: Supplementary file 1 [file ijerph-19-13158-s001.zip › Table S1.pdf]

State	Lindane use kg/cropland		Thyroid Cancer IR (2019)		
	1992	2007	Male and Female	Male	Female
Alabama	0.51	0.05	9.4	4.6	13.8
Alaska	NA	NA	12.1	6.8	17.9
Arizona	0.005 (1996)	0.1 (2001)	11.7	6.2	17.2
Arkansas	0.007 (1993)	0.003	12.8	6.7	18.7
California	0.23	0.002 (2005)	13.0	6.9	19.0
Colorado	0.0006	0.02	12.7	8.5	17.2
Connecticut	0.57 (1995)	0.06	16.7	9.1	24.1
Delaware	0.16 (1995)	0.8	14.2	8.0	20.1
Florida	0.12	0.0003 (2005)	12.5	6.7	18.2
Georgia	2.33	0.07	11.2	6.3	15.7
Idaho	0.51	0.06 (2004)	12.7	7.1	18.4
Illinois	0.007 (1993)	0.02	13.5	7.0	19.8

Indiana	0.015	0.02	10.5	5.9	15.0
Iowa	0.03 (1993)	0.03	14.5	8.8	20.4
Kansas	0.04	0.01	14.0	7.5	20.7
Kentucky	0.04	0.02	14.0	6.7	21.2
Louisiana	0.005 (1993)	0.07 (2006)	14.1	7.3	20.7
Maine	NA	NA	15.9	8.0	23.6
Maryland	0.007	0.25	12.3	6.6	17.7
Massachusetts	0.34 (1995)	0.03	14.3	7.8	20.4
Michigan	0.38	0.01	11.6	6.5	16.6
Minnesota	0.02	0.01	10.6	6.3	15.0
Mississippi	0.04	0.01	8.0	4.5	11.4
Missouri	0.01 (1993)	0.03	11.6	6.2	16.6
Montana	0.17 (1993)	0.0001 (2006)	14.0	9.9	18.3
Nebraska	0.02	0.04	14.7	8.4	21.2

Nevada	0.0007	0.0007 (2005)	NA	NA	NA
New Hampshire	0.34 (1995)	0.04	12.0	5.3	18.7
New Jersey	0.61	0.008	17.2	9.3	25.0
New Mexico	0.002	0.002	16.4	9.8	22.9
New York	0.15	0.1	19.6	10.8	28.1
North Carolina	0.004	0.17	10.6	5.4	15.5
North Dakota	0.1 (1993)	0.0024 (2006)	19.6	10.1	30.1
Ohio	0.05	0.03	14.8	7.3	22.1
Oklahoma	0.01	0.0015	12.7	7.1	18.3
Oregon	0.03	0.03 (2005)	11.7	5.5	17.8
Pennsylvania	0.27	0.002	15.9	9.0	22.7
Rhode Island	0,34 (1995)	0.02	15.9	9.1	22.5
South Carolina	0.04	0.12	9.7	5.7	13.5
South Dakota	0.02 (2004)	0.005	16.2	9.2	23.7
Tennessee	0.02	0.01	10.5	5.6	15.1

Texas	0.04	0.004	11.9	6.4	17.4
Utah	0.38	0.38	16.5	9.0	24.2
Vermont	0.46 (1995)	0.05	14.5	6.9	22.0
Virginia	0.007	0.11	11.0	5.3	16.6
Washington	0.0003	0.006	11.6	5.7	17.6
West Virginia	0.009	0.006	16.3	7.6	24.9
Wisconsin	0.1	0.04	12.8	7.5	18.2
Wyoming	0.03	0.012 (2006)	14.8	8.1	22.0
