# Supplementary figures and images for: Correlation between Lindane Use and the Incidence of Thyroid Cancer in the United States: An Ecological Study
Source: Int J Environ Res Public Health. 2022 Oct 13;19(20):13158. doi: 10.3390/ijerph192013158 (PMC9602460; doi:10.3390/ijerph192013158)

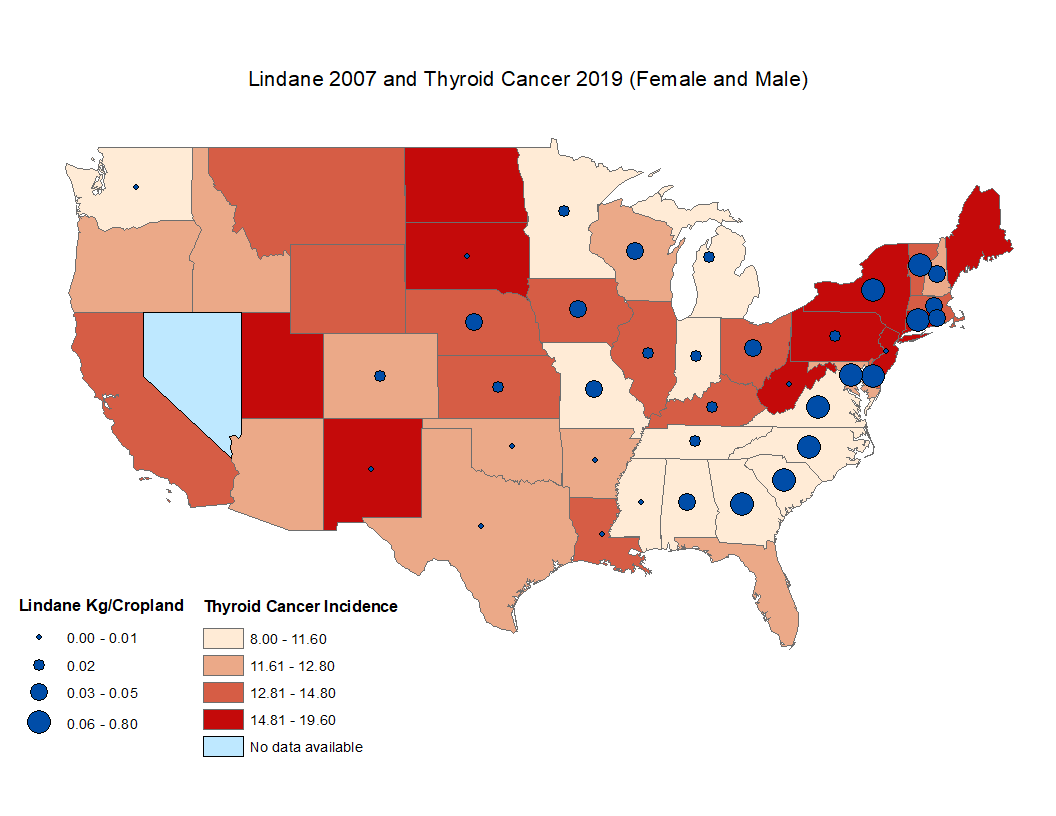

Supplement: Supplementary file 1 [file ijerph-19-13158-s001.zip › Figure S1.png]

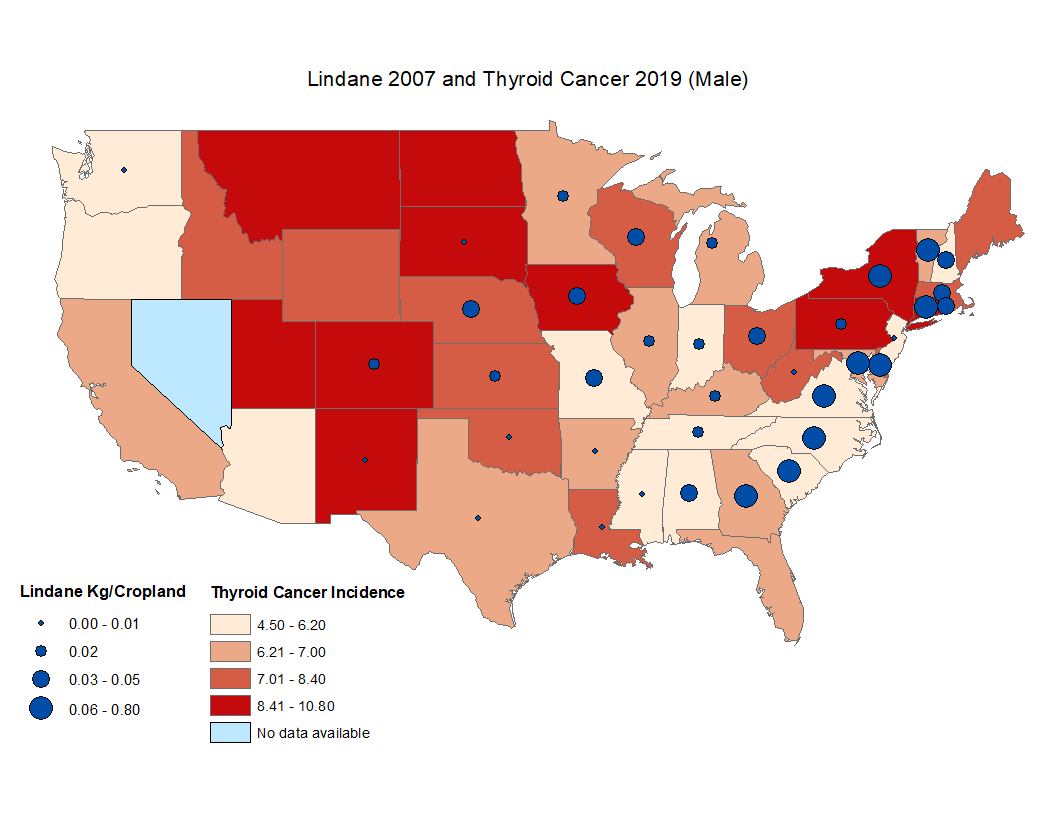

Supplement: Supplementary file 1 [file ijerph-19-13158-s001.zip › Figure S2.png]

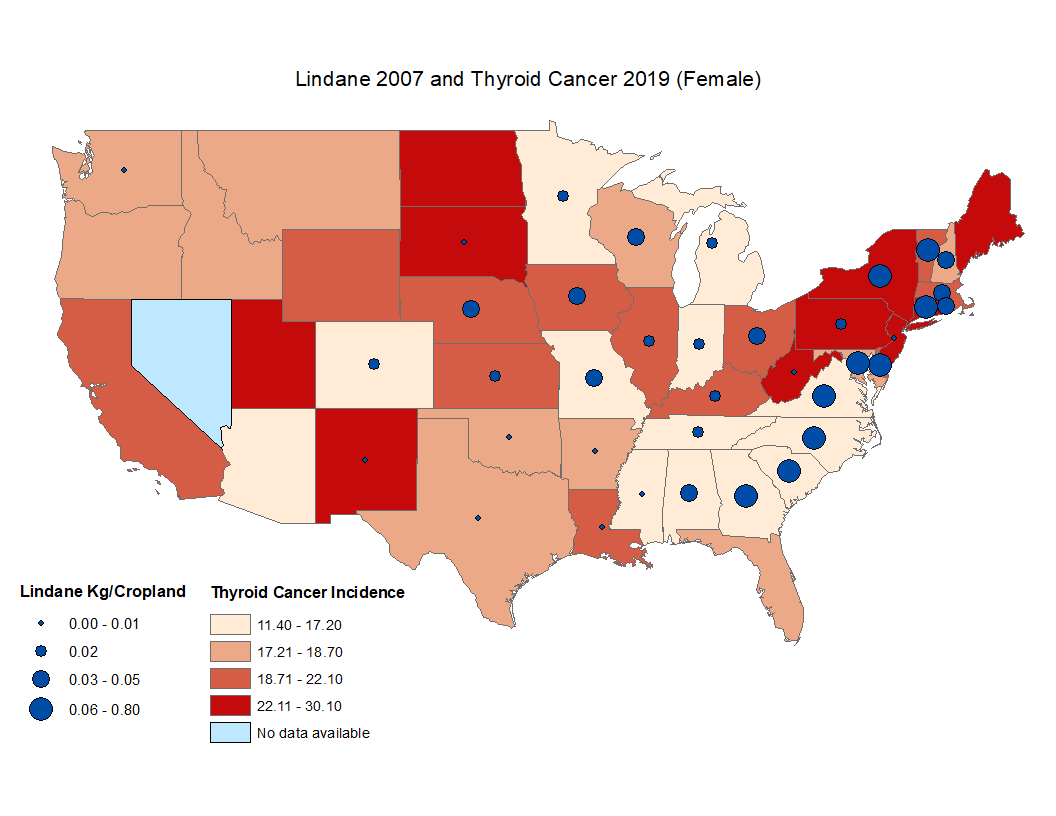

Supplement: Supplementary file 1 [file ijerph-19-13158-s001.zip › Figure S3.png]

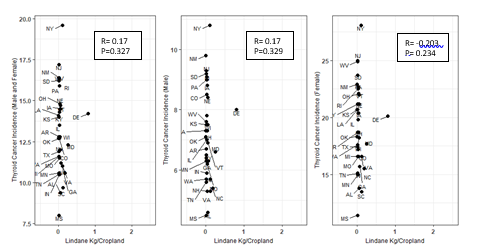

Supplement: Supplementary file 1 [file ijerph-19-13158-s001.zip › Figure S4.PNG]
